# Multi-Omics Epigenetic Landscape Unveils Regulatory Mechanisms Underlying Heterosis in Sheep Muscle Development

**DOI:** 10.3390/ani16071112

**Published:** 2026-04-04

**Authors:** Jiangbo Cheng, Dan Xu, Huibin Tian, Xiaoxue Zhang, Liming Zhao, Runan Zhang, Jianlin Wang, Jinyu Xiao, Fadi Li, Weimin Wang, Deyin Zhang

**Affiliations:** 1 State Key Laboratory of Herbage Improvement and Grassland Agro-Ecosystems, Key Laboratory of Grassland Livestock Industry Innovation, Ministry of Agriculture and Rural Affairs, Engineering Research Center of Grassland Industry, Ministry of Education, College of Pastoral Agriculture Science and Technology, Lanzhou University, Lanzhou 730020, China; chengjb2023@lzu.edu.cn (J.C.); 15045093462@163.com (D.X.); tianhb@lzu.edu.cn (H.T.); zlmfxy1807285865@163.com (L.Z.); zhangrunan818@163.com (R.Z.); jlwang@lzu.edu.cn (J.W.); xiaojycy@lzu.edu.cn (J.X.); lifd@lzu.edu.cn (F.L.); wangweimin@lzu.edu.cn (W.W.); 2 College of Animal Science and Technology, Gansu Agricultural University, Lanzhou 730070, China; zhangxx@gsau.edu.cn

**Keywords:** sheep, hybrid vigor, breed-specific regulatory elements, *PPARGC1A*

## Abstract

Hybridization effectively enhances breeding efficiency and significantly boosts sheep productivity. However, the epigenetic mechanisms underlying the superior production performance of crossbreds remain largely elusive. In this study, Hu sheep were crossbred with Suffolk rams used as the paternal line. We integrated RNA-seq, ATAC-seq, and CUT&Tag (H3K4me3, H3K4me1, H3K27ac, and H3K27me3) techniques to characterize epigenetic regulatory differences in the longissimus dorsi muscle between Hu sheep (HU) and crossbred progeny (SH). Phenotypic and transcriptomic analyses revealed that SH crossbred sheep exhibited superior growth performance (*p* < 0.05), and the upregulated genes in the Apelin signaling pathway were significantly correlated with eye muscle area (*p* < 0.05). Utilizing a Hidden Markov Model, we annotated 15 distinct chromatin states in both HU and SH sheep, systematically characterizing the dynamic epigenomic landscapes across the two breeds. In contrast to SH sheep, the genome of HU sheep exhibited enrichment of repressive chromatin modifications typified by H3K27me3. Strong active enhancers (EnhA) were significantly enriched within upregulated genes in SH. A total of 1862 SH-specific and 691 HU-specific EnhA elements were characterized in this study. Motif analysis revealed that SH-specific EnhA were enriched for myogenic MEF2 family motifs (*p* < 0.05), which promote muscle and vascular development. By integrating multi-omics data, we constructed a putative regulatory network potentially modulated by SH-specific enhancers, identifying *CMKLR1*, *PPARGC1A*, and *TLE3* as the core hub genes. Collectively, this study provides a robust data resource, identifying candidate genes and regulatory elements associated with crossbreeding-related muscle phenotypes.

## Simple Summary

Crossbreeding serves as an efficient strategy to rapidly enhance sheep meat production. Crossing Suffolk rams with Hu ewes significantly improves growth traits. However, the regulatory mechanisms underlying this heterosis remain unclear. This study investigated the epigenetic differences between crossbred sheep and purebred Hu sheep. Our results reveal that the up-regulated genes in crossbred sheep are significantly enriched in the Apelin signaling pathway. We annotated 15 distinct epigenetic regulatory elements across the sheep longissimus dorsi muscle. Functional regulatory elements of sheep were enriched in the up-regulated genes of crossbred sheep, while a stronger repressive regulatory signal was present in the Hu sheep genome. By integrating multi-omics data, we identified a gene network regulated by crossbred sheep-specific enhancers. Collectively, a proportion of the upregulated genes in crossbred sheep are modulated by crossbred-specific enhancers, providing candidate genes and a useful resource for the breeding of high-yielding mutton sheep.

## 1. Introduction

Sheep (*Ovis aries*) are among the most widely distributed livestock species globally. Mutton is rich in protein and unsaturated fatty acids, serving as a vital source of high-quality animal protein [1,2]. The Hu (HU) sheep is a premier indigenous breed in China, celebrated for exceptional maternal traits such as year-round estrus, early sexual maturity, high prolificacy, and excellent lactation performance [3]. However, its overall production efficiency is fundamentally limited by a slow growth rate and a low dressing percentage [4,5]. Crossbreeding is a primary strategy in livestock breeding, as crossbred progeny frequently exhibit production performance superior to that of their parents. This approach allows for the effective combination of desirable traits from distinct breeds or lines [6]. Recognized globally as the largest meat breed in terms of frame size and body weight, the Suffolk sheep is extensively utilized as a terminal sire in meat sheep production systems [7]. Notably, the Suffolk × HU crossbreds display exceptional growth performance, characterized by significantly increased body weights and average daily gain relative to their maternal HU counterparts. Although this crossbreeding strategy effectively improves mutton production efficiency, the fundamental regulatory mechanisms, especially the genetic and epigenetic landscapes orchestrating this advantageous phenotype, remain largely unresolved. Given that Hu sheep serve as the foundational maternal breed in commercial sheep production systems, utilizing this maternal line as a rigorous baseline to evaluate the molecular improvements of crossbred progeny constitutes the core objective of current breeding programs. Consequently, existing studies have focused their comparative analyses on crossbred progeny and purebred Hu sheep, aiming to elucidate how crossbreeding improves the muscle development traits of this maternal breed [8,9,10].

Currently, the formation of heterosis is mainly attributed to the dominance and overdominance hypotheses [11]. Accumulating evidence indicates that the genetic basis of heterosis is extraordinarily complex [12,13]. Heterosis arises as a consequence of transcriptionally regulated gene expression in hybrids, whereas its phenotypic performance is not governed by a simplistic integration of genes from both parents [14]. With the rapid advancement of epigenomic technologies, the epigenetic hypothesis of heterosis has gradually emerged. Investigations into DNA methylation and non-coding RNAs have provided novel perspectives and strategies for deciphering the underlying mechanisms of hybrid vigor. In plants, the expression of most miRNAs is repressed in hybrids compared with parental inbred lines, leading to increased global levels of transcription and translation, which may constitute the primary mechanism underlying F1 heterosis [15]. Genomic methylation, which typically represses gene expression, can directly or indirectly contribute to advantageous phenotypic outcomes in hybrids [16]. This epigenetic regulatory mechanism underlying heterosis has been documented in species such as *Arabidopsis* [17] and soybean [18]. Furthermore, genomic chromatin accessibility plays a role in the regulation of gene expression underlying growth trait heterosis [19]. Although existing studies have utilized transcriptomic [20], proteomic [8], and metabolomic [9] approaches in skeletal muscle to identify a series of downstream candidate molecules associated with the growth traits of crossbred sheep, the upstream molecular mechanisms driving these transcriptional alterations remain to be fully elucidated. Although previous studies have demonstrated the existence of differential DNA methylation networks in sheep muscle, the analysis of histone modifications and chromatin accessibility is also critical for elucidating the active epigenetic regulatory mechanisms that drive these transcriptional networks [10].

Public projects such as the Functional Annotation of Animal Genomes (FAANG) have facilitated the annotation of epigenetic functional elements in livestock genomes [21], providing valuable resources for deciphering the epigenetic mechanisms underlying heterosis. However, the annotation of regulatory elements in the sheep genome is currently limited to the HU sheep breed, with no corresponding annotation available for hybrid progeny [22]. This hinders the understanding of the epigenetic mechanisms underlying the superior production traits of crossbred sheep. Therefore, this study integrated RNA-seq, ATAC-seq, and CUT&Tag profiles for 4 distinct histone modifications from the muscle tissues of maternal HU sheep and SH crossbreds to identify candidate epigenetic regulatory features associated with muscle development, thereby constructing a foundational multi-omics resource. Specifically, we focused on 4 canonical histone modifications: H3K4me3 and H3K27ac, which demarcate active promoters and enhancers; H3K4me1, a well-established enhancer mark; and H3K27me3, which mediates transcriptional repression [23]. Leveraging this dataset, we systematically annotated the genome-wide epigenetic regulatory elements across both breeds, thereby dissecting the transcriptomic divergences and their underlying upstream epigenetic regulatory mechanisms. Collectively, these results provide robust data support for future in-depth investigations into the epigenetic mechanisms underlying the muscle phenotypes of crossbred sheep.

## 2. Materials and Methods

### 2.1. Animals and Sample Collection

In this study, purebred male Hu (HU) and male crossbred Suffolk × HU (SH) sheep were utilized as experimental subjects. A total of 30 HU and 30 SH lambs born on the same day were selected. All the sheep used in this study were purchased from a commercial sheep farm and raised at the experimental base of Lanzhou University. All animals were weaned at 56 days of age. During the entire experimental period, the sheep were provided ad libitum access to feed and water, and uniform housing and environmental conditions were maintained throughout the study. Body weight was measured at 100 and 120 days of age following a 12-h fasting period. At 120 days of age, three HU and three SH sheep were randomly selected and humanely euthanized. The longissimus dorsi muscle tissues were immediately harvested, rinsed with pre-chilled PBS to remove residual blood, and snap-frozen in liquid nitrogen for subsequent experiments. Concurrently, the eye muscle area of the carcasses was measured.

### 2.2. Library Preparation and High-Throughput Sequencing

In total, 26 libraries were constructed, consisting of 3 biological replicates per breed for RNA-seq, and 2 biological replicates per breed for ATAC-seq and CUT&Tag assays.

For RNA-seq, total RNA was extracted from muscle tissue using TRIzol reagent (Invitrogen, Carlsbad, CA, USA). The libraries were constructed using the VAHTS Total RNA-seq Library Prep Kit for Illumina^®^ (Vazyme, Nanjing, China), and the library construction process was strictly followed according to the kit instructions. Following construction, the libraries were initially quantified using a Qubit 3.0 Fluorometer (Invitrogen, Carlsbad, CA, USA). Insert size and overall quality were subsequently evaluated on an Agilent 2100 Bioanalyzer (Agilent Technologies, Santa Clara, CA, USA). Once the effective concentration was confirmed to exceed 10 nM, the samples were subjected to high-throughput sequencing on the Illumina NovaSeq 6000 platform (Illumina, San Diego, CA, USA).

For ATAC-seq, approximately 5 mg of the frozen tissue sample was placed into a 5-mL glass Dounce homogenizer and homogenized in 1 mL of ice-cold PBS buffer. The resulting homogenate was subsequently filtered, and nuclear morphology was evaluated under a light microscope. Libraries were constructed using the Hyperactive ATAC-Seq Library Prep Kit for Illumina^®^ (Vazyme, Nanjing, China) with approximately 20,000 nuclei per reaction, strictly following the manufacturer’s protocol. The resulting libraries were quantified with a Qubit 3.0 Fluorometer (Invitrogen, Carlsbad, CA, USA) and subsequently normalized. Insert size and overall library integrity were evaluated using an Agilent 4200 Bioanalyzer (Agilent Technologies, Santa Clara, CA, USA). Upon passing quality control, the qualified libraries were sequenced on the Illumina NovaSeq 6000 platform (Illumina, San Diego, CA, USA).

For CUT&Tag, cell nuclei were extracted using the same method as ATAC-seq. Libraries were constructed using the Hyperactive Universal CUT&Tag Assay Kit for Illumina^®^ Pro (Vazyme, Nanjing, China) with approximately 20,000 nuclei per reaction, strictly following the manufacturer’s protocol. Primary antibodies against H3K4me3, H3K27ac, H3K4me1, and H3K27me3 were used in the assay. The library inspection and sequencing processes were consistent with ATAC-seq.

### 2.3. Raw Sequencing Data Analysis

In total, 26 novel datasets were generated in this study, comprising 6 RNA-seq, 4 ATAC-seq, and 16 CUT&Tag datasets (H3K4me3, H3K4me1, H3K27ac, and H3K27me3). The detailed bioinformatics pipelines are described as follows:

For RNA-seq data, raw sequencing data were filtered using fastp (version 0.23.2) [24] to remove adapter sequences and low-quality reads. The resulting clean reads were aligned to the sheep reference genome (Oar_rambouillet_v1.0 (GCF_002742125.1)) using HISAT2 (version 2.2.1) [25]. Samtools (version 1.17) [26] was used to filter and sort the aligned data to generate a BAM file. FeatureCounts (version 2.0.3) [27] was used to quantify the genes in the reference genome. R software (version 4.3.3) was used to perform differential analysis via the DESeq2 package (version 1.42.0), while functional enrichment analysis of genes was carried out using the clusterProfiler package (version 4.10.1) [28].

For ATAC-seq and CUT&Tag data, raw sequencing data were filtered using fastp (version 0.23.2) to remove adapter sequences and low-quality reads. Bowtie2 (version 2.4.4) [29] was used to align clean reads to the sheep reference genome. Samtools (version 1.17) and Picard (version 2.27.5) were utilized to sort the aligned reads, filter low-quality alignments, and remove PCR duplicates. The callpeak function of MACS2 (version 2.2.7.1) [30] was used to call peaks. Peak annotation was performed utilizing the ChIPseeker package (version 1.38.0) [31] in R (version 4.3.3). For data visualization, biological replicates were pooled using the merge function of SAMtools (version 1.17), and the merged files were converted into bigwig format using the bamCoverage tool in deeptools (version 3.5.1) [32]. The computeMatrix and plotProfile functions of deepTools (version 3.5.1) were used to calculate the average signal of epigenetic marks using bigwig as the input file. IGV (version 2.16.2) was used to visualize the bigwig files. BAM files were converted to BED format using the bamtobed utility in bedtools (version 2.30.0). To assess global data consistency, the genome was divided into 500-bp bins, and Pearson correlation coefficients for read distributions were calculated across RNA-seq, ATAC-seq, and CUT&Tag datasets. Heatmaps were generated using the ComplexHeatmap package (version 2.18.0) in R (version 4.3.3).

Publicly available whole-genome bisulfite sequencing (WGBS) data were retrieved from the SRR18578183 dataset. Initial quality control and adapter trimming of the raw sequences were executed via Trim Galore (v0.6.10). The resulting high-quality reads were mapped to the reference genome utilizing Bismark (v0.24.2). PCR duplicates were discarded employing the MarkDuplicates tool from the Picard (version 2.27.5). Cytosine methylation statuses at CpG dinucleotides were parsed using the bismark_methylation_extractor module. Mean methylation fractions across defined distinct chromatin states were calculated with the map command in bedtools (version 2.30.0).

### 2.4. Chromatin State Annotation

ATAC-seq and CUT&Tag datasets from both HU and SH sheep were integrated to perform chromatin state segmentation using ChromHMM (version 1.25) [33]. A 15-state model was employed for the genome-wide annotation. Based on the distinct enrichment patterns of epigenetic marks within each state, these chromatin states were functionally defined as Strongly active promoters/transcripts (TssA), Weak promoters/transcripts (TssAWk), Flanking active TSS without ATAC (TssAHet), Transcribed at gene (TxFlnk), Transcribed region without ATAC (TxFlnkHet), Weak transcribed at gene (TxFlnkWk), Strong active enhancer (EnhA), Active enhancer no ATAC (EnhAHet), Medium enhancer with ATAC (EnhAMe), Weak active enhancer (EnhAWk), Poised enhancer (EnhPois), ATAC island (ATAC_Is), Bivalent/poised TSS (TssBiv), Repressed polycomb (Repr), and Quiescent (Qui). The average methylation level for each chromatin state was calculated using the map function of bedtools (version 2.30.0). The enrichment folds of different chromatin states in differentially expressed genes were calculated using previously reported methods [34].

### 2.5. Identification of Breed-Specific Regulatory Elements (BSR)

To identify BSR, a consensus set of non-redundant EnhA regions across both breeds was generated using bedtools (version 2.30.0). The EnhA peaks from the two HU and two SH replicates were then intersected with this consensus set, applying a binary scoring system where an overlap was scored as 1 (presence) and a non-overlap as 0 (absence). Non-redundant EnhA regions scoring 1 in both replicates of a single breed, but 0 in the other, were strictly defined as BSRs. Conversely, regions scoring 1 in both breeds were classified as shared EnhA. We performed motif identification in BSR EnhA elements using the findMotifsGenome.pl script from the HOMER software package (version 4.11) [35].

### 2.6. Statistical Analysis

All statistical analyses were performed using the R statistical environment (version 4.3.3). Pearson correlation coefficients were calculated via the native cor() function. Furthermore, phenotypic differences between groups were evaluated using the Wilcoxon rank-sum test. For both the differential and functional enrichment analyses, statistical significance was determined using nominal *p*-values, with a significance threshold set at *p* < 0.05.

### 2.7. Quantitative Real-Time-PCR (qRT-PCR) Validation of Differentially Expressed Genes

Total RNA was extracted using the AG RNAex Pro Reagent (Accurate Biotechnology, Changsha, China). RNA integrity and concentration were evaluated via agarose gel electrophoresis and a NanoDrop 2000 spectrophotometer (Thermo Fisher Scientific, Waltham, MA, USA), respectively. Subsequently, cDNA was synthesized using the Evo M-MLV RT Kit with gDNA Clean for qPCR (Accurate Biotechnology, Changsha, China) following the manufacturer’s standard protocols. The qRT-PCR assays were conducted using the SYBR Green Premix Pro Taq HS qPCR Kit (Accurate Biotechnology, Changsha, China), with thermal cycling conditions performed as previously described [22]. Relative target gene expression was calculated using the 2^−ΔΔCt^ method [36], with *UXT* employed as the internal reference. All primer sequences used in this study are detailed in Table S6. All qRT-PCR assays were performed using three biological replicates, with four technical replicates per sample.

## 3. Results

### 3.1. Data Summary

**Figure 1:**
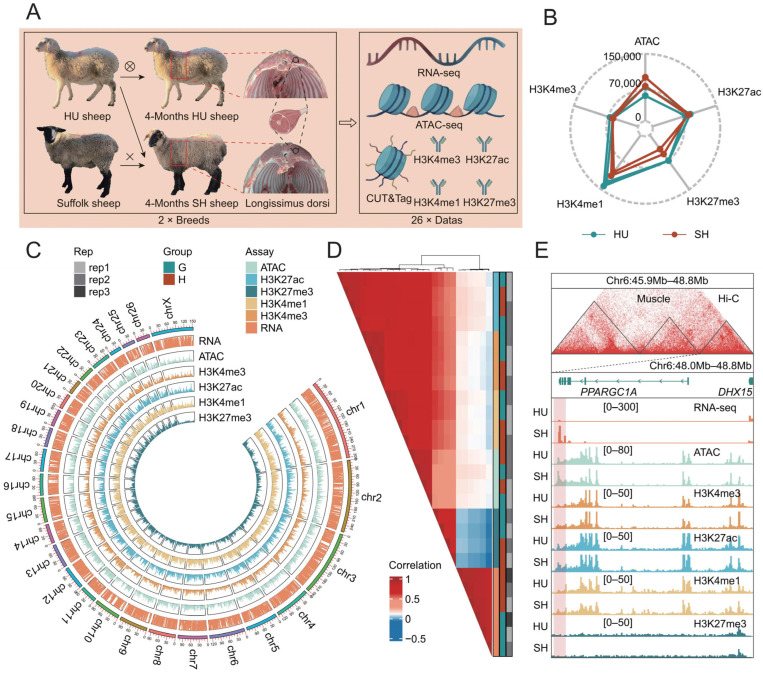
Construction of an epigenetic atlas of the longissimus dorsi muscle in sheep. (**A**) Schematic overview of the experimental design and datasets. Longissimus dorsi muscle tissues were collected from 120-day-old HU and SH sheep and subjected to RNA-seq (*n* = 3), ATAC-seq (*n* = 2), and CUT&Tag (H3K4me3, H3K4me1, H3K27ac, and H3K27me3) sequencing (*n* = 2). (**B**) The number of peaks for different epigenetic markers in the longissimus dorsi muscle of HU and SH sheep. (**C**) Circos plot illustrating the genome-wide coverage of transcriptome reads and the chromosomal distribution of epigenetic peaks. (**D**) Heatmap of Pearson correlation coefficients across different omics assays, breeds, and biological replicates, calculated based on 500-bp genomic windows. (**E**) IGV visualization of epigenetic profiles within the TAD encompassing the *PPARGC1A* gene.

To investigate the changes in transcriptional and epigenetic regulatory elements underlying muscle development between SH crossbred sheep (Suffolk × HU sheep) and their maternal HU sheep, and to identify the key differentially regulated genes, we generated an integrated epigenomic dataset using the longissimus dorsi muscle tissues of HU sheep and SH sheep. This dataset consists of RNA-seq libraries, ATAC-seq libraries, and CUT&Tag assays targeting four histone modifications (H3K4me3, H3K27ac, H3K4me1, and H3K27me3) (Figure 1A). We obtained a total of 2.75 billion raw reads, and after filtering, we obtained 2.73 billion high-quality clean reads that were mapped to the reference genome, with an average alignment rate of 98.43% (Table S1). Analysis of the fragment size distribution revealed that all epigenomic libraries exhibited a characteristic nucleosome-free region peak and a mono-nucleosomal peak (Figure S1A). As expected, H3K4me3 showed the highest enrichment around the TSS, where the remaining active regulatory marks (H3K27ac, H3K4me1, and ATAC-seq) also formed distinct peaks (Figure S1B). Furthermore, according to ENCODE guidelines, the library complexity metrics (PBC1 > 0.9, PBC2 > 3, and NRF > 0.7) demonstrated that the sequencing data quality was highly robust for downstream analyses (Table S2).

### 3.2. Epigenomic Regulatory Landscapes of Ovine Longissimus Dorsi Muscle

Across the two breeds, we identified an average of 77,565, 83,514, 58,675, 121,687, and 61,209 peaks from the ATAC-seq, H3K27ac, H3K27me3, H3K4me1, and H3K4me3 datasets, respectively (Table S2). The corresponding average peak lengths were 424.72, 562.10, 500.49, 751.71, and 755.31 bp. Furthermore, the fraction of reads in peaks (FRiP) analysis revealed that all assays yielded scores greater than 0.2, with the sole exception of the repressive histone mark H3K27me3 (Table S2). Notably, we observed that SH sheep exhibited a greater number of ATAC-seq peaks, whereas HU sheep possessed more H3K27me3 peaks (Figure 1B). Utilizing the identified peaks, we constructed a genome-wide epigenomic landscape of the sheep longissimus dorsi muscle, revealing a comprehensive distribution of distinct epigenetic marks across the chromosomes (Figure 1C). Genomic annotation of these peaks demonstrated that H3K4me3 was predominantly enriched in promoter regions, whereas H3K27me3 was primarily localized to distal intergenic regions (Figure S1C). Furthermore, all assessed regulatory elements exhibited a substantial proportion of peaks within intronic regions. Based on the read distribution across genomic windows, we evaluated the correlations among different assays, breeds, and biological replicates. The results demonstrated that datasets were primarily clustered according to assay type. Within each assay category, biological replicates from different breeds clustered together. Furthermore, active regulatory marks (H3K4me3, H3K27ac, H3K4me1, and ATAC-seq) exhibited strong positive correlations with one another and a weak positive correlation with the transcriptome. Conversely, the repressive regulatory mark (H3K27me3) was negatively correlated with transcriptional expression (Figure 1D, Table S3). To elucidate the importance of the epigenetic regulatory landscape in muscle development, we used the *PPARGC1A* gene as a representative example, which serves as a master regulator of mitochondrial biogenesis and myofiber type transition in skeletal muscle. By integrating publicly available sheep Hi-C data [37], we visualized the epigenetic regulatory landscape within the topologically associating domain (TAD) encompassing *PPARGC1A* (Figure 1E). Our results revealed that SH sheep exhibited upregulated *PPARGC1A* expression accompanied by higher ATAC-seq and H3K27ac signals, whereas HU sheep displayed stronger H3K27me3 enrichment. These findings underscore that alterations in epigenetic regulatory elements are strongly associated with the differential gene expression induced by Suffolk-HU crossbreeding.

### 3.3. Growth Performance and Transcriptomic Profiles of HU and SH Sheep

**Figure 2:**
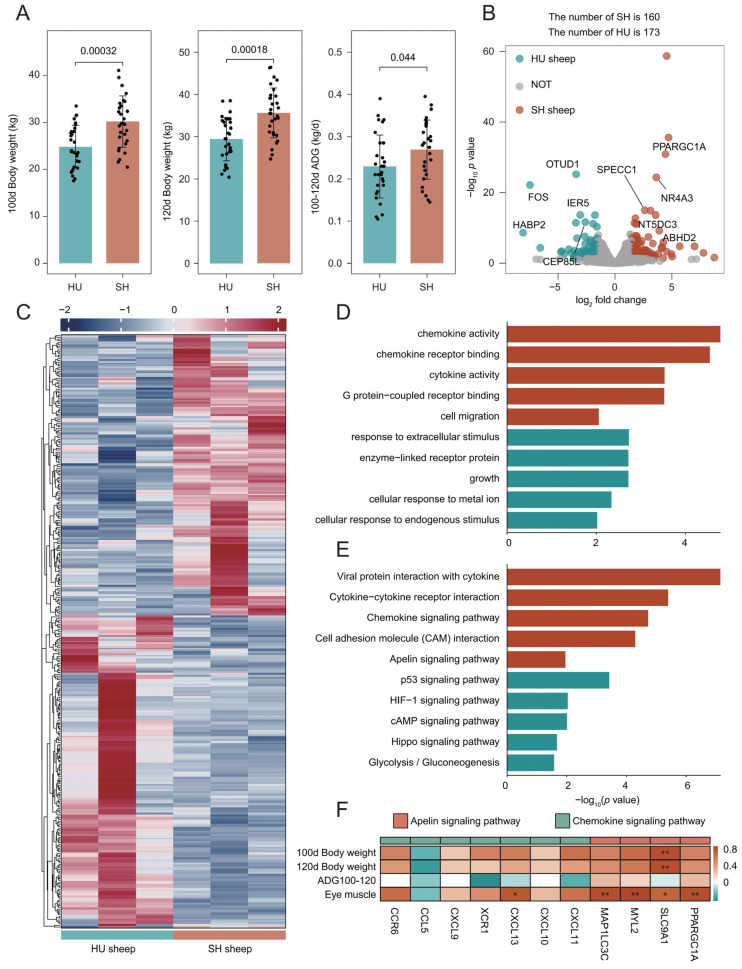
Phenotypic and transcriptomic divergence between HU and SH sheep. (**A**) Statistical analysis of body weights at 100 and 120 days of age, and average daily gain from 100 to 120 days, evaluated via the Wilcoxon rank-sum test. (**B**) Volcano plot showing DEGs identified using the DESeq2 package (*p* < 0.05). Red and blue indicate genes upregulated in SH and HU sheep, respectively. (**C**) Heatmap illustrating the expression profiles of the DEGs. (**D**,**E**) GO (**D**) and KEGG (**E**) enrichment analyses of the DEGs, with red and blue denoting enrichment results for SH- and HU-upregulated genes. (**F**) Spearman correlation heatmap between phenotypic traits and SH-upregulated genes enriched in the Chemokine and Apelin signaling pathways. * *p* < 0.05; ** *p* < 0.01.

We compared the growth performance between the HU and SH sheep populations. As illustrated in Figure 2A, the body weights of SH sheep at 100 and 120 days of age were significantly higher than those of HU sheep. Furthermore, the average daily gain (ADG) from 100 to 120 days was also significantly greater in the SH cohort (*p* < 0.05).

To investigate the molecular basis underlying these phenotypic differences, we analyzed the transcriptome of longissimus dorsi muscle from 120-day-old sheep and identified 13,756 expressed genes with transcripts per million (TPM) > 1. Using thresholds of |Fold Change| > 2 and *p*-value < 0.05, we identified a total of 333 differentially expressed genes (DEGs) (Table S4). Among these, 160 genes were upregulated in the SH sheep, while 173 genes were upregulated in the HU sheep (Figure 2B). Hierarchical clustering analysis illustrated the expression patterns of DEGs, showing a clear divergence between the two breeds alongside high consistency among biological replicates within each group (Figure 2C). The mRNA levels of DEGs were quantified by qRT-PCR. As presented in Figure S2, the expression profiles were consistent with the RNA-seq results, confirming the credibility of the transcriptome analysis.

Gene Ontology (GO) enrichment analysis of the DEGs revealed that the SH-upregulated genes were primarily enriched in terms such as ‘chemokine activity’, ‘chemokine receptor binding’, and ‘G protein-coupled receptor binding’ (Figure 2D). In contrast, the HU-upregulated genes were mainly enriched in categories including ‘response to extracellular stimulus’, ‘cellular response to endogenous stimulus’, and ‘cellular response to metal ion’ (Figure 2D). KEGG pathway enrichment analysis revealed that the upregulated genes in SH sheep were significantly enriched in the ‘Chemokine signaling pathway’, ‘Cytokine-cytokine receptor interaction’, and ‘Apelin signaling pathway’ (Figure 2E). Conversely, the upregulated genes in HU sheep were primarily enriched in pathways such as the ‘p53 signaling pathway’ and ‘HIF-1 signaling pathway’. Given the superior growth performance of the SH sheep, we directed our attention to the specific DEGs enriched in the Chemokine and Apelin signaling pathways, both of which are intimately involved in myogenesis and metabolic homeostasis (Figure 2E). We conducted a correlation analysis between the DEGs enriched in these two pathways and the phenotypic traits. The results revealed that *SLC9A1* was significantly positively correlated with eye muscle area and body weights at 100 and 120 days of age (*p* < 0.05) (Figure 2F). Furthermore, *PPARGC1A*, *MYL2*, and *MAP1LC3C* exhibited a highly significant positive correlation with eye muscle area (*p* < 0.01), alongside positive associations with other phenotypes (Figure 2F).

### 3.4. Characterization of Chromatin States in Ovine Longissimus Dorsi Muscle

**Figure 3:**
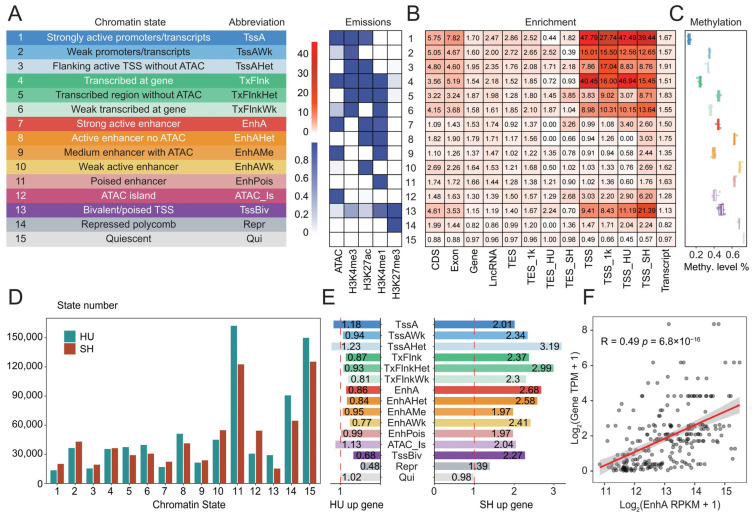
Chromatin state annotation in the ovine longissimus dorsi muscle. (**A**) Nomenclature, abbreviations, and emission probabilities of epigenetic marks for the defined chromatin states. (**B**) Genomic enrichment profiles of the 15 chromatin states. (**C**) Average DNA methylation levels across distinct chromatin states. (**D**) The number of non-redundant chromatin states in the HU and SH genomes. (**E**) Enrichment of chromatin states within the genomic regions of HU- and SH-upregulated genes. (**F**) Pearson correlation analysis between the expression levels (TPM) of SH-upregulated genes and the signal intensities (RPKM) of their proximal EnhA elements.

To systematically annotate the regulatory elements, we employed a multivariate Hidden Markov Model (ChromHMM) to integrate five epigenetic profiles (ATAC-seq, H3K4me3, H3K27ac, H3K4me1, and H3K27me3) from the longissimus dorsi muscle, successfully defining an optimal 15-state chromatin model for both the HU and SH sheep genomes (Figure S3). These 15 chromatin states were subsequently annotated as promoters (TssA, TssAWk, TssAHet, and TssBiv), transcribed regions (TxFlnk, TxFlnkHet, and TxFlnkWk), enhancers (EnhA, EnhAHet, EnhAMe, EnhAWk, and EnhPois), accessible islands (ATAC_Is), repressed regions (Repr), and quiescent regions (Qui) (Figure 3A). Promoter states exhibited the highest enrichment directly at the TSS (transcription start site), whereas the TssAHet state showed greater enrichment in the 1-kb flanking regions of the TSS than at the TSS. Interestingly, ATAC_Is and TssBiv displayed higher enrichment at the TSSs of SH-upregulated genes compared to those of HU-upregulated genes (Figure 3B). Based on publicly available DNA methylation data from the sheep longissimus dorsi muscle [37], we evaluated the methylation levels across different chromatin states. As expected, the results revealed that TssA exhibited the lowest methylation levels, whereas the Repr and Qui states displayed the highest methylation levels (Figure 3C).

Overall, we identified 624,028 and 576,570 non-redundant chromatin states in the HU and SH sheep, respectively, with average lengths ranging from 332.56 to 21,656.20 bp (Table S5). These functional regulatory elements covered 11.65% and 9.65% of the HU and SH genomes, respectively. Notably, the SH sheep exhibited higher counts and proportions of TssA and EnhA compared to the HU sheep, whereas the HU sheep possessed a greater abundance and proportion of TssBiv and Repr states (Table S5 and Figure 3D). This suggests that the expression of a subset of genes in the HU sheep might be transcriptionally repressed. To investigate whether the genomic regions of DEGs were enriched for regulatory elements, we quantified the enrichment of 15 distinct chromatin states in DEGs. The analysis revealed that multiple chromatin states were significantly enriched in the SH-upregulated genes, whereas no such significant enrichment was observed in the HU-upregulated genes (Figure 3E). Notably, TssAHet, TxFlnkHet, and EnhA exhibited the highest enrichment levels in the SH-upregulated genes. To further explore the regulatory relationship between EnhA and DEGs, we performed a linear correlation analysis between the expression levels of DEGs and the H3K27ac signals of EnhA (Figure 3F). The results demonstrated a significant positive correlation (*p* < 0.05), indicating that the expression of these DEGs is potentially modulated by EnhA elements.

### 3.5. Breed-Specific Analysis of EnhA

**Figure 4:**
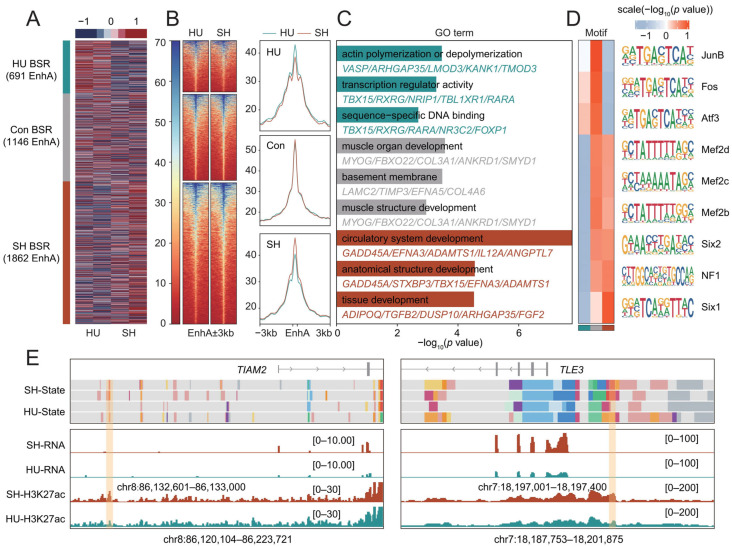
Functional characterization of breed-specific regulatory elements (BSR). (**A**) Heatmaps of H3K27ac signal intensities across the identified BSR EnhA. (**B**) Distribution of H3K27ac signals across BSR EnhA regions and their ±3-kb flanking regions. The red and blue lines represent SH and HU sheep, respectively. (**C**) Gene Ontology (GO) enrichment analysis of the target genes of BSR EnhA. Blue, red, and grey indicate HU-specific, SH-specific, and shared EnhA, respectively. (**D**) Significance (*p*-values) of enriched transcription factor binding motifs within the BSR EnhA. (**E**) IGV visualization of representative target genes putatively regulated by BSR EnhA.

To investigate breed-specific regulatory elements (BSR), we identified 691 and 1862 BSR EnhA in HU and SH sheep, respectively (Figure 4A). The H3K27ac signals in the BSR showed the corresponding trend (Figure 4B). Gene Ontology (GO) enrichment analysis revealed that the target genes of HU-specific BSRs were primarily enriched in functions such as ‘transcription regulator activity’ and ‘actin polymerization or depolymerization’ (Figure 4C). In contrast, the target genes of SH-specific BSRs were significantly enriched in ‘circulatory system development’, ‘anatomical structure development’, and ‘tissue development’ (Figure 4C). Meanwhile, the target genes regulated by the shared EnhAs were predominantly involved in fundamental myogenic processes, including ‘muscle organ development’ and ‘muscle structure development’ (Figure 4C). Concurrently, we performed an enrichment analysis of transcription factor binding motifs. The results revealed that motifs for core myogenic transcription factors, such as MEF2C, MEF2D, and MEF2B, were significantly enriched in both the shared and SH BSR (Figure 4D). Collectively, these results indicate that BSR EnhA are putative regulators of key genes involved in muscle development. For instance, *TIAM2* [38] and *TLE3* [39], two well-known genes implicated in muscle development and differentiation, both harbor SH-specific BSR EnhAs in their upstream regions (Figure 4E). Consistently, the expression levels of these two genes were significantly higher in the SH sheep compared to the HU sheep (*p* < 0.05).

### 3.6. Identification of Candidate Genes for Muscle Development via Integrated Multi-Omics

**Figure 5:**
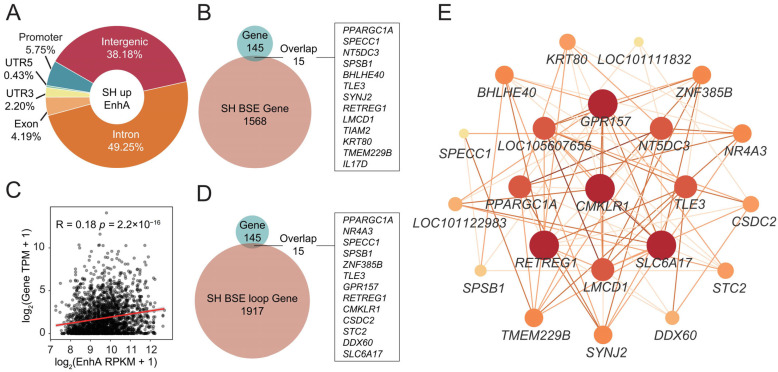
Construction of a regulatory network through multi-omics integration. (**A**) Genomic distribution of SH-specific BSR EnhA. (**B**) Overlap between the putative target genes of SH BSR EnhAs and SH-upregulated genes, highlighting non-LOC genes. (**C**) Pearson correlation analysis between the signal intensities of SH BSR EnhAs and the expression levels of their loop-linked putative target genes. (**D**) Overlap between the loop-linked putative target genes of SH BSR EnhAs and SH-upregulated genes, showing non-LOC genes. (**E**) Co-expression network constructed based on the identified overlapping genes. Only edges meeting the thresholds of R > 0.8 and *p* < 0.05 are retained. Darker edge colors indicate stronger correlations, and larger node sizes represent a higher topological degree.

We primarily focused on the SH BSR EnhA. Genomic annotation revealed that these elements are predominantly distributed within intronic and intergenic regions (Figure 5A). Integration with the transcriptomic data identified 15 overlapping genes between those associated with SH BSR EnhAs and the SH-upregulated DEGs, including *PPARGC1A*, *SPECC1*, and *NT5DC3* (Figure 5B). Furthermore, considering the potential for distal regulation by enhancers, we utilized publicly available Hi-C data from the sheep longissimus dorsi muscle to construct chromatin loop structures [37]. Correlation analysis between the EnhA signals and target gene expression at the paired loop anchors demonstrated a significant correlation, although it was relatively weaker than that observed for proximal regulation (Figure 5C). We identified 15 overlapping genes between the distal target genes of SH BSR EnhA and the DEGs, including *PPARGC1A*, *NR4A3*, and *SPECC1* (Figure 5D). Taken together, these SH BSR EnhA accounted for 17.24% (25/145) of the upregulated DEGs in the SH sheep. An expression correlation network was constructed using the 25 identified candidate genes, retaining edges with R > 0.8 and *p*-value < 0.05 (Figure 5E). The results revealed that *CMKLR1*, *PPARGC1A*, and *TLE3* shared significant positive correlations with a large number of candidate genes, indicating that they likely act as crucial hub genes within this regulatory network that potentially contribute to SH sheep muscle development.

## 4. Discussion

The HU sheep is a premier indigenous breed in China, renowned for its high prolificacy and robust stress resistance; however, its application in meat production is limited by low meat yield [40]. Crossbreeding is a widely adopted strategy to enhance livestock productivity. As a dominant and one of the largest meat sheep breeds globally, the Suffolk sheep is frequently utilized for genetic improvement [10]. Our present results demonstrated that both the growth rate and eye muscle area of the Suffolk × HU (SH) crossbred sheep were significantly higher than those of the maternal HU sheep, thereby effectively improving mutton production efficiency. However, the multilayered molecular mechanisms, particularly the epigenetic regulatory landscapes underlying this superior muscle phenotype, have remained largely elusive. In this study, by integrating transcriptomic, epigenomic (ATAC-seq and CUT&Tag), and 3D genomic architectures, we present a comprehensive and high-resolution regulatory map of the ovine longissimus dorsi muscle. Our findings reveal that BSR serve as critical regulatory hubs. Overall, this study provides a valuable resource for the efficient utilization of heterosis in sheep breeding.

Elucidating the regulatory mechanisms underlying heterosis in complex livestock traits is a prerequisite for its efficient exploitation. Phenotypic variations are fundamentally driven by gene expression, a process in which epigenetic regulatory elements play a pivotal role [34,41]. Consequently, based on five crucial epigenetic profiles, we characterized the global chromatin landscapes of both HU and SH sheep to explore how 14 distinct functional regulatory elements are associated with transcriptional networks in the SH crossbreds. Compared to the SH crossbreds, the maternal HU genome exhibited a profoundly stronger transcriptional repression. This repressive landscape is highlighted by a greater genomic abundance of Repr and TssBiv states, closely associated with the extensive deposition of the repressive histone modification H3K27me3 [42]. The TssBiv state represents a bivalent chromatin domain characterized by the coexistence of the activating H3K4me3 and the repressive H3K27me3 histone marks [43]. Upon the loss of the H3K27me3 mark, these bivalent promoters resolve into an active state, initiating gene transcription [44]. This suggests that a subset of genes in the HU breed may be epigenetically repressed, thereby restricting muscle differentiation and development. In contrast, crossbreeding with Suffolk sheep might diminish the repressive H3K27me3 signals, thereby potentially facilitating gene expression [45].

Directing our focus toward the DEGs identified in the transcriptome, we observed a significant enrichment of crucial functional regulatory elements, notably TssA and EnhA, within the SH-upregulated genes. Furthermore, by integrating the H3K27ac signals at EnhA regions with the DEG expression profiles, we found supporting evidence for the potential proximal regulatory role of EnhA in modulating gene expression, a finding that aligns well with previous reports [46,47]. While previous studies have established that EnhA exhibit strong tissue specificity [22,48], our results revealed a specific enrichment of EnhAs within the SH-upregulated genes. This observation led us to propose that EnhAs also display distinct breed specificity. Consequently, we identified 1862 SH-specific and 691 HU-specific BSR EnhAs, which exhibited remarkably different functional trajectories. Based on the GO analysis, the shared EnhAs predominantly maintain basal myogenic processes by targeting genes associated with muscle differentiation, such as *MYOG* [49]. Notably, the target genes of SH BSR EnhA were uniquely enriched in pathways such as ‘circulatory system development’ and ‘anatomical structure development’. Given that muscle fiber hypertrophy strictly requires an abundant supply of nutrients from the circulatory system [50], we hypothesize that the potentially enhanced maturation of the vascular network in SH sheep might effectively support their accelerated muscle development. Furthermore, the biological efficacy of enhancers intrinsically relies on the recruitment of specific transcription factors [51]. Consistent with this, our motif analysis revealed a significant enrichment of MEF2 family (MEF2B, MEF2C, and MEF2D) binding sites within both the shared and SH-specific BSR EnhA. Recognized as the master regulators of myogenesis, the MEF2 proteins act as the central hub of the myogenic regulatory network [52,53]. Interestingly, the HU BSR EnhA exhibited a profound enrichment for the AP-1 transcription factor complex (including JunB, Fos, and Atf3) [54]. Given that the AP-1 signaling pathway can inhibit muscle cell division and promote fatty acid metabolism, it consequently restricts the rapid proliferation of myoblasts [55]. Consistent with the epigenetic EnhA-associated transcriptional programs, our transcriptomic analysis identified the Apelin and Chemokine signaling pathways as key downstream pathways significantly enriched among the SH-upregulated genes. The Apelin signaling pathway is a positive regulator of skeletal muscle development, exerting a dual role in stimulating microvascular angiogenesis and enhancing energy metabolism to support muscle growth [56]. The present dataset demonstrated that all DEGs enriched in the Apelin signaling pathway exhibit a significant positive correlation with the eye muscle area. The p53 and HIF-1 signaling pathways were significantly enriched among the upregulated genes in HU sheep. The p53 pathway exerts a negative regulatory effect on myogenesis, while enhanced HIF-1 signaling promotes glycolysis [57,58]. This reflects the maintenance of cellular homeostasis within the muscle of HU sheep.

We also considered the distal regulatory role of EnhA. Loop-mediated interactions between EnhA and its target genes were significantly correlated, albeit with a weaker correlation than that of proximal regulatory enhancers, a finding consistent with previous studies [48]. By integrating the proximal and distal target genes of the SH BSR EnhA, we revealed that 17.24% of the upregulated genes in SH sheep can be potentially associated with EnhA-mediated regulation. Within this network, several critical hub genes were identified, including *CMKLR1*, *PPARGC1A*, and *LC6A17*. Notably, *PPARGC1A*, a key gene enriched in the Apelin signaling pathway, acts as a crucial transcriptional coactivator governing mitochondrial biogenesis and energy metabolism [59]. Furthermore, *PPARGC1A* is a crucial regulatory factor that drives the conversion of muscle fibers from Type II to Type I [60]. Consistent with this, *MYH7B*, a canonical marker gene for Type I muscle fibers, was significantly upregulated in SH sheep, which further supports the results (Table S4). Its enhancer-associated transcriptional activation in the SH crossbreds potentially supports the metabolic demands required for muscle fiber growth. Meanwhile, previously reported findings on *CMKLR1* are consistent with the results of the transcriptomic pathway analysis, highlighting the critical role of chemokine signaling in regulating muscle regeneration [61]. Our findings suggest that the regulatory network centered on *CMKLR1* provides promising candidate genes responsible for heterosis in the muscle development of SH sheep. These genetic resources can be applied to facilitate the breeding of high-yield mutton sheep through genomic selection, thereby accelerating the genetic improvement and enhancing the production efficiency of meat sheep.

However, several limitations of the present study must be acknowledged. First, to address the practical needs of ongoing breeding programs, the comparative design of this study focused on the crossbred progeny and their maternal HU sheep. While this design offers valuable implications for understanding maternal-line improvement, the lack of paternal data restricts the comprehensive assessment of classical heterosis. Second, the relatively small sample size employed for multi-omics analyses limited the statistical power of the study. Third, although we integrated publicly available Hi-C and DNA methylation datasets, these data were not generated from our experimental groups, which may introduce unaccounted-for variation associated with differences in developmental stages. Furthermore, the analytical results obtained in this study have not undergone functional validation, and further verification at the cellular and population levels is still required.

## 5. Conclusions

In summary, this study provides a useful multi-omics resource by constructing the epigenetic and transcriptional landscape of the sheep longissimus dorsi muscle. By systematically integrating these datasets, we successfully identified candidate genes and regulatory elements associated with crossbreeding-related transcriptional differences between the HU and SH sheep. Notably, the *CMKLR1* regulatory network identified through multi-omics integration emerges as a promising candidate framework for deciphering the heterosis underlying sheep muscle development. These findings identify valuable candidate genes and their upstream epigenetic regulatory elements, providing a foundational resource for future research on heterotic traits in crossbred sheep.

## Data Availability

The data used in the study analyses can be made available by the corresponding author on reasonable request.

## Supplementary Materials

The following supporting information can be downloaded at: https://www.mdpi.com/article/10.3390/ani16071112/s1, Figure S1: (A) Insertion fragment length distribution of ATAC and CUT&Tag. (B) Enrichment of different apparent signals in the TSS and TES regions. (C) Annotation distribution of peaks of ATAC and CUT&Tag. Figure S2: Validation of RNA-Seq by qRT-PCR analysis. Figure S3: Determination of the optimal chromatin state model. The heatmap illustrates the maximum Pearson correlation coefficients between each state in the full model (y-axis) and its best-matching counterpart in simpler models (x-axis). The upper panel displays a line plot depicting the median correlation values for each evaluated model relative to the full model. The final selected model is highlighted by a black box. Table S1: Overview of sheep RNA-seq, ATAC-seq and CUT&Tag data. Table S2: Peak statistics of ATAC-seq and CUT&Tag. Table S3: Pearson correlation values of assays, breeds, and two biological replicates. Table S4: Differentially expressed genes in HU and SH. Table S5: The number, length, and coverage of 15 chromatin states. Table S6: Details of primer sequences used for qRT-PCR.

## Abbreviations

The following abbreviations are used in this manuscript:

HU	Hu
SH	Suffolk × HU crossbreds
TAD	topologically associating domain
DEGs	differentially expressed genes
BSR	breed-specific regulatory elements
ADG	average daily gain
TSS	transcription start site
TPM	transcripts per million
GO	Gene Ontology
KEGG	Kyoto Encyclopedia of Genes and Genomes
FRiP	fraction of reads in peaks
NRF	non-redundant fraction
RPKM	reads per kilobase of transcript, per million mapped reads
