# Supplementary material for: Multi-Omics Epigenetic Landscape Unveils Regulatory Mechanisms Underlying Heterosis in Sheep Muscle Development
Source: Animals (Basel). 2026 Apr 4;16(7):1112. doi: 10.3390/ani16071112 (PMC13072424; doi:10.3390/ani16071112)
Supplement: Supplementary file 1 [file animals-16-01112-s001.zip › animals_Supplementary Figure_V2.pdf]

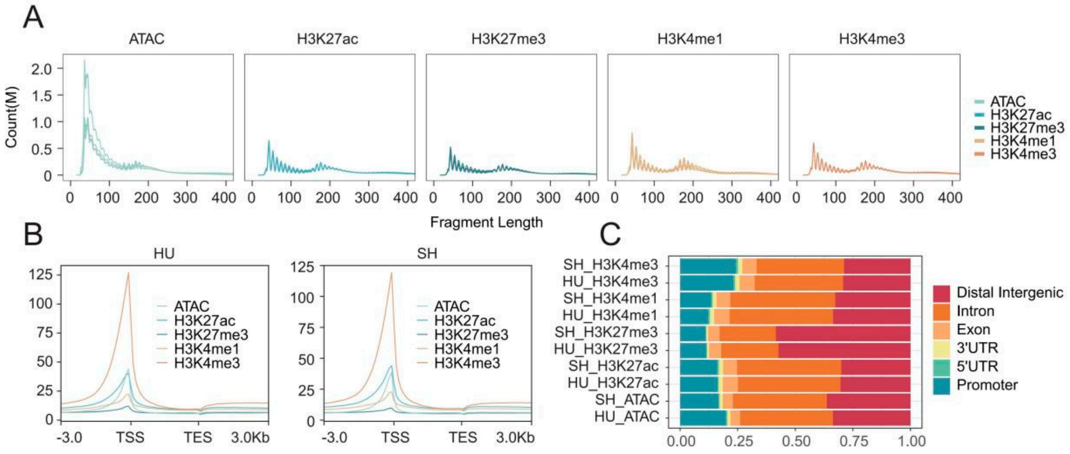


**Supplementary Figure S1.** (A) Insertion fragment length distribution of ATAC and CUT&Tag. (B) Enrichment of different apparent signals in the TSS and TES regions. (C) Annotation distribution of peaks of ATAC and CUT&Tag.

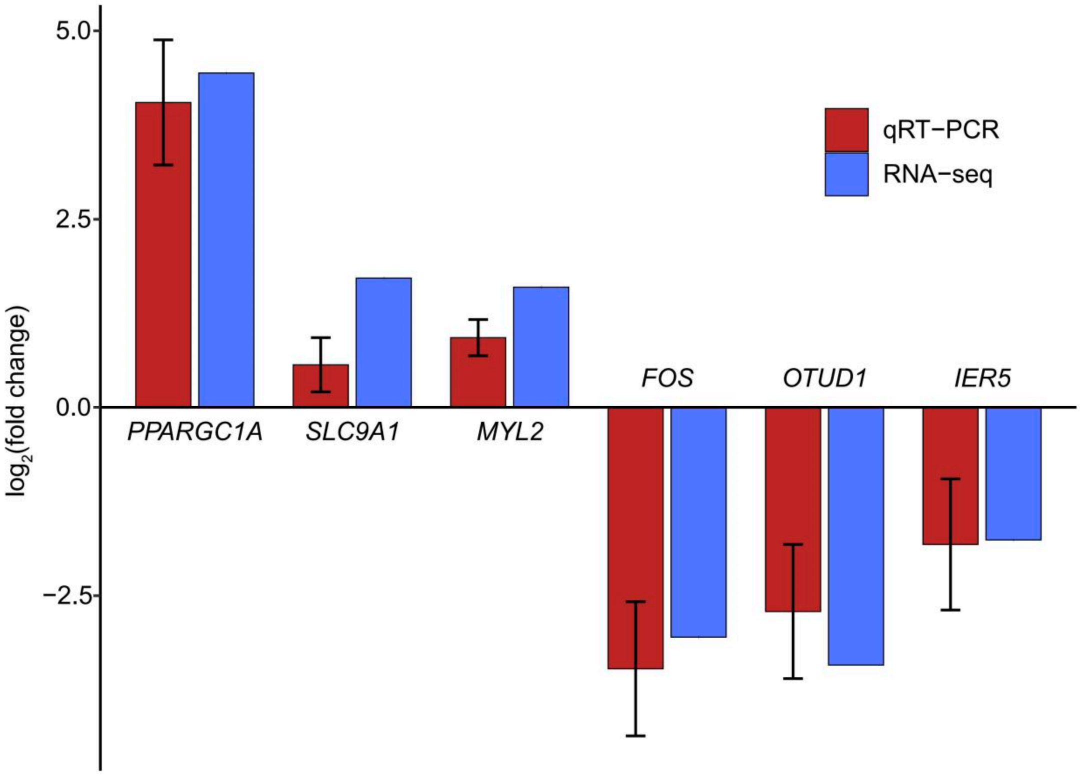


**Supplementary Figure S2.** Validation of RNA-Seq by qRT-PCR analysis.

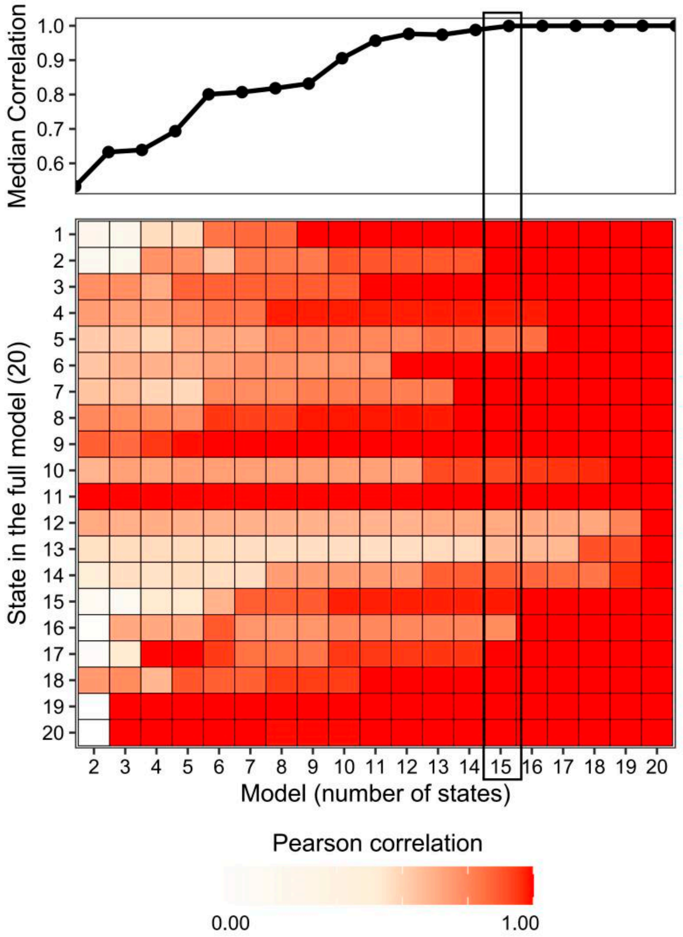


**Supplementary Figure S3.** Determination of the optimal chromatin state model. The heatmap illustrates the maximum Pearson correlation coefficients between each state in the full model (y-axis) and its best-matching counterpart in simpler models (x-axis). The upper panel displays a line plot depicting the median correlation values for each evaluated model relative to the full model. The final selected model is highlighted by a black box.
